# Biochemical characterization and cellular imaging of a novel, membrane permeable fluorescent cAMP analog

**DOI:** 10.1186/1471-2091-9-18

**Published:** 2008-06-25

**Authors:** Daniela Moll, Anke Prinz, Cornelia M Brendel, Marco Berrera, Katrin Guske, Manuela Zaccolo, Hans-Gottfried Genieser, Friedrich W Herberg

**Affiliations:** 1 University of Kassel, Department of Biochemistry, Heinrich-Plett-Strasse 40, 34132 Kassel, Germany; 2 University of Kassel, Department of Physical Chemistry, Heinrich-Plett-Strasse 40, 34132 Kassel, Germany; 3 University of Glasgow, University Avenue, Glasgow G12 8QQ, Scotland, UK; 4 BIOLOG Life Science Institute, Flughafendamm 9a, P.O. Box 107125, Bremen, Germany

## Abstract

**Background:**

A novel fluorescent cAMP analog (8-[Pharos-575]- adenosine-3', 5'-cyclic monophosphate) was characterized with respect to its spectral properties, its ability to bind to and activate three main isoenzymes of the cAMP-dependent protein kinase (PKA-Iα, PKA-IIα, PKA-IIβ) *in vitro*, its stability towards phosphodiesterase and its ability to permeate into cultured eukaryotic cells using resonance energy transfer based indicators, and conventional fluorescence imaging.

**Results:**

The Pharos fluorophore is characterized by a Stokes shift of 42 nm with an absorption maximum at 575 nm and the emission peaking at 617 nm. The quantum yield is 30%. Incubation of the compound to RIIα and RIIβ subunits increases the amplitude of excitation and absorption maxima significantly; no major change was observed with RIα. *In vitro *binding of the compound to RIα subunit and activation of the PKA-Iα holoenzyme was essentially equivalent to cAMP; RII subunits bound the fluorescent analog up to ten times less efficiently, resulting in about two times reduced apparent activation constants of the holoenzymes compared to cAMP. The cellular uptake of the fluorescent analog was investigated by cAMP indicators. It was estimated that about 7 μM of the fluorescent cAMP analog is available to the indicator after one hour of incubation and that about 600 μM of the compound had to be added to intact cells to half-maximally dissociate a PKA type IIα sensor.

**Conclusion:**

The novel analog combines good membrane permeability- comparable to 8-Br-cAMP – with superior spectral properties of a modern, red-shifted fluorophore. GFP-tagged regulatory subunits of PKA and the analog co-localized. Furthermore, it is a potent, PDE-resistant activator of PKA-I and -II, suitable for *in vitro *applications and spatial distribution evaluations in living cells.

## Background

Fluorescent nucleotides have become widely utilized tools in basic research [1], and the number of corresponding reports on their use in cellular systems is vast. However studies involving the second messenger cAMP have not kept pace, and studies using fluorescently tagged cAMP analogs are still limited.

Early reports mainly used nucleobase-modified analogs where the purine ring system was part of the fluorophore, such as 1, N^6^- etheno-cAMP [2], 2- aza- 1, N^6^- etheno-cAMP [3] or the cyclic phosphate of 2- aminopurine riboside [4]. However, these compounds are far from being optimal with regard to membrane permeability, cAMP-dependent protein kinase (PKA) binding affinity and stability towards phosphodiesterases (PDEs). Further more, the fluorophores lack brilliance and possess unfavorable spectral properties, e.g., excitation is to be performed in the UV range, which can be harmful to intact cells and monitoring of the relatively short emission wavelengths is often disturbed by intrinsic fluorescent components of the cell.

For studies involving PDEs, anthraniloyl [5]- and methylanthraniloyl- modified [6] cAMP (MANT-cAMP) have been introduced, where the ribose 2- position carries a fluorescent reporter group. Since the 2'- modification render these structures unable to activate PKA, the MANT group has been linked to the positions 6 [7] and 8 [8] of the adenine nucleobase as well (MABA-cAMP). According to corresponding lipophilicity data (log k_w_), cyclic nucleotides with MANT modification have improved membrane permeability and better PDE-resistance (at least 6- and 8- modified structures), but are still suboptimal with respect to their spectral properties. The same holds true for cAMP modified with the NBD fluorophore (8-[2-[(7-Nitro-4-benzofurazanyl)amino]ethyl]thio]adenosine-3',5'-cyclic monophosphate; 8-NBD-cAMP) [6].

Fluorescein and rhodamine have been attached to the 8- position of cAMP as well [9], however, in spite of the improved spectral properties of these dyes, both conjugates are not membrane permeable due to an additional charge within the dye moiety. Unfortunately, nearly all modern fluorescent dye structures contain positive or negative charges which support the electronic push/pull mechanism of the respective chromophore but render the corresponding conjugates rather polar, especially when attached to nucleotides with their polar phosphate groups. Even cAMP conjugates with state-of-the-art dyes such as Cy3 [10], Evoblue, and Bodipy^® ^[11,12], which have excellent spectral properties, fail to pass cellular membranes, and require invasive application techniques like patch clamp or microinjection or the osmotic lysis of pinocytic vesicles [13] and are mainly utilized *in vitro *assays. Importantly, if these dyes are connected to the 2'- ribose moiety, the resulting conjugates will not bind to and activate PKA anymore. Finally, phosphate-modified caged cAMP analogs have been described, which – upon photo-activation – release cAMP together with fluorescent coumarines [14,15].

Thus, in spite of quite a number of different fluorescent variants of cAMP, these structures have only limited application scopes, and presently no analog is available that offers improved properties in all important aspects mentioned, and which could be used with intact cells for tracking or intracellular imaging experiments.

The main effector enzyme of cAMP is the cAMP-dependent protein kinase (PKA), which reversibly phosphorylates substrate proteins. Protein kinases and their counter players, phosphatases and cAMP-degrading PDEs, are key regulatory enzymes in eukaryotic cells. PKA is a multi-substrate enzyme mediating the majority of the known effects of cAMP by regulating the activity of proteins involved in signal transduction, energy metabolism, cell proliferation, and differentiation [16]. In the absence of cAMP, all PKA isoforms consist of two regulatory (R) and two catalytic subunits (C) that form an inactive tetramer. Binding of at total of four cAMP molecules to the two tandem cAMP binding sites of each R subunit promotes the dissociation of the holoenzyme complex and leads to the release of the now active C subunits phosphorylating target proteins in the cytosol or in the nucleus [17]. Main isoforms of the C subunit are Cα, Cβ, Cγ and PrKX, and several minor isoforms have been identified at least at cDNA level. In human, four different isoforms of the R subunit (RIα, RIβ, RIIα, RIIβ) have been identified [18].

Besides PKA and its corresponding signaling pathway, cAMP addresses additional cellular targets such as cyclic nucleotide-dependent ion channels (cyclic nucleotide gated ion channel and hyperpolarization-activated cyclic nucleotide-modulated channel) as well as the exchange protein directly activated by cAMP (Epac), which are worthwhile objects for evaluation with fluorescent analogs [19,20].

In view of the importance of the cAMP messenger system, improved tools for a more detailed investigation of functions and receptor distribution would be quite helpful. Thus, in this study, the properties of a novel commercially available conjugate of cAMP with the Pharos dye, 8-[Pharos-575]- adenosine-3', 5'-cyclic monophosphate (8-[ϕ-575]-cAMP) and the corresponding free dye were investigated and analyzed physico-chemically (spectral properties, stability), with respect to PKA-RI and -RII subunit binding as well as holoenzyme activation *in vitro *and in living cells, PDE-resistance, and cellular uptake.

## Results and Discussion

### Photochemical characterization of the Pharos dye

**Figure 1:**
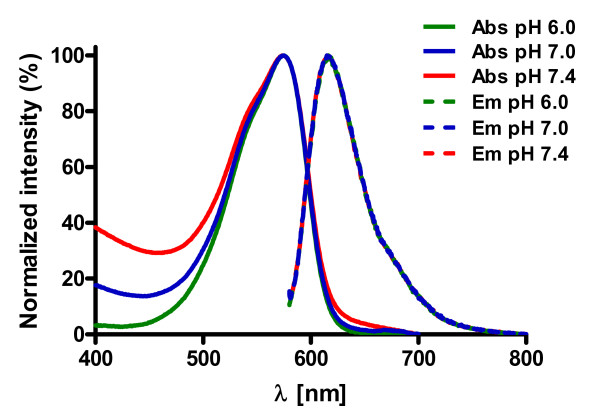
**Fluorescence absorption and emission spectra of the Pharos dye**. The absorption maxima of the Pharos dye dissolved in buffer with the indicated pH values is at 575 nm, whereas the emission maxima are at 617 nm. All spectra exhibit a large Stokes shift of 42 nm.

**Figure 2:**
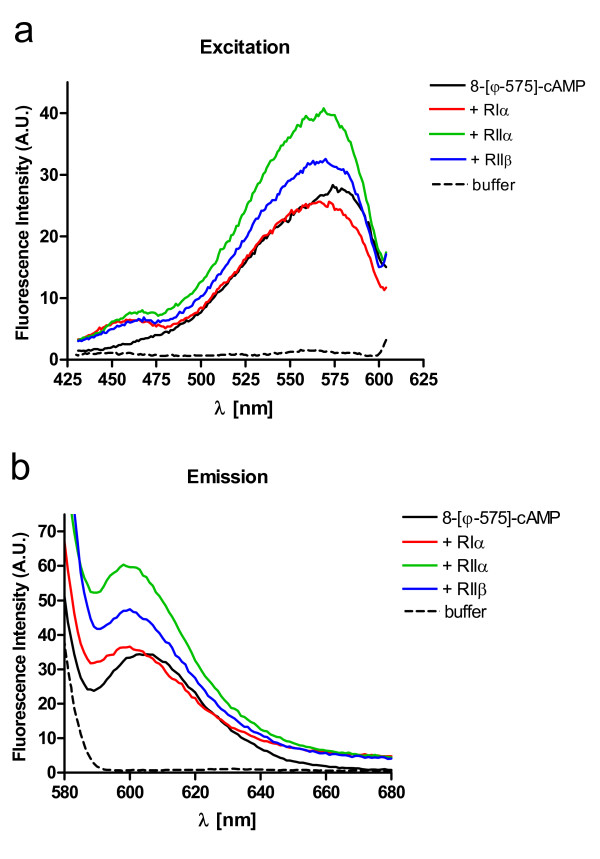
**Fluorescence spectra of 8-[ϕ-575]-cAMP bound to PKA R subunits**. 150 nM 8-[ϕ-575]-cAMP was incubated with or without fourfold molar excess of PKA R subunits as indicated in the figure. The excitation spectra (a) were detected at Em 617 nm and Ex 610 nm-430 nm; the emission spectra (b) were detected at Ex 575 nm and Em 680 nm-580 nm. The experiments were repeated four times with similar results.

First we determined the photo-chemical properties of the free Pharos dye. The Stokes shift at pH 6.0, pH 7.0 and pH 7.4 was 42 nm resulting from an absorption maximum at 575 nm and an emission maximum at 617 nm (Fig. 1; ε_575nm _= 15,650). 8-[ϕ-575]-cAMP behaved similarly with an excitation maximum at 577 nm and emission maximum at 605 nm at pH 7.0 (Fig. 2, and data not shown).

To calculate the relative quantum yield ϕ of the Pharos dye the absorption (500 nm to 640 nm) and fluorescence (λ_ex _= 553 nm, λ_em _ranging from 558 nm to 800 nm) spectra of several Pharos dye concentrations (640 nM to 64.2 μM) were recorded using quinine sulfate as a standard [21,22]. The determination of the relative quantum yield is generally accomplished by plotting the integrated fluorescence intensity versus the absorbance at the excitation wavelength (see Additional file 1, panel a). By comparing the slope of the linear regression (see Additional file 1, panel b) with that of a standard substance (quinine sulfate), the relative quantum yield of the Pharos dye was determined to be 30%.

### Spectral properties of 8-[ϕ-575]-cAMP upon binding to PKA R-subunits

8-[ϕ-575]-cAMP was incubated with or without a fourfold molar excess of RIα, RIIα and RIIβ subunits (Fig. 2). The excitation spectra (a) were detected at Em 617 nm and Ex 610 nm-430 nm; the emission spectra (b) were detected at Ex 575 nm and Em 680 nm – 580 nm. Upon addition of RIIα and RIIβ subunits we observed an increase of the amplitude of excitation and emission maxima, whereas the interaction of the analog with RIα protein had no significant effect. Furthermore, a slight shift of the excitation and emission spectra to shorter wavelengths (Ex = 570 nm; Em = 600 nm) was found with all three R subunits.

### 8-[ϕ-575]-cAMP binding to PKA R subunits and activation of PKA

It has been shown that cAMP analogs, modified in position 8 of the adenine nucleobase, are powerful activators of protein kinase A, and even rather bulky substituents are accepted here. In addition, all analogs modified with fluorophores of high molecular weight and steric demands were reported to be potent PKA agonists[7].

**Figure 3:**
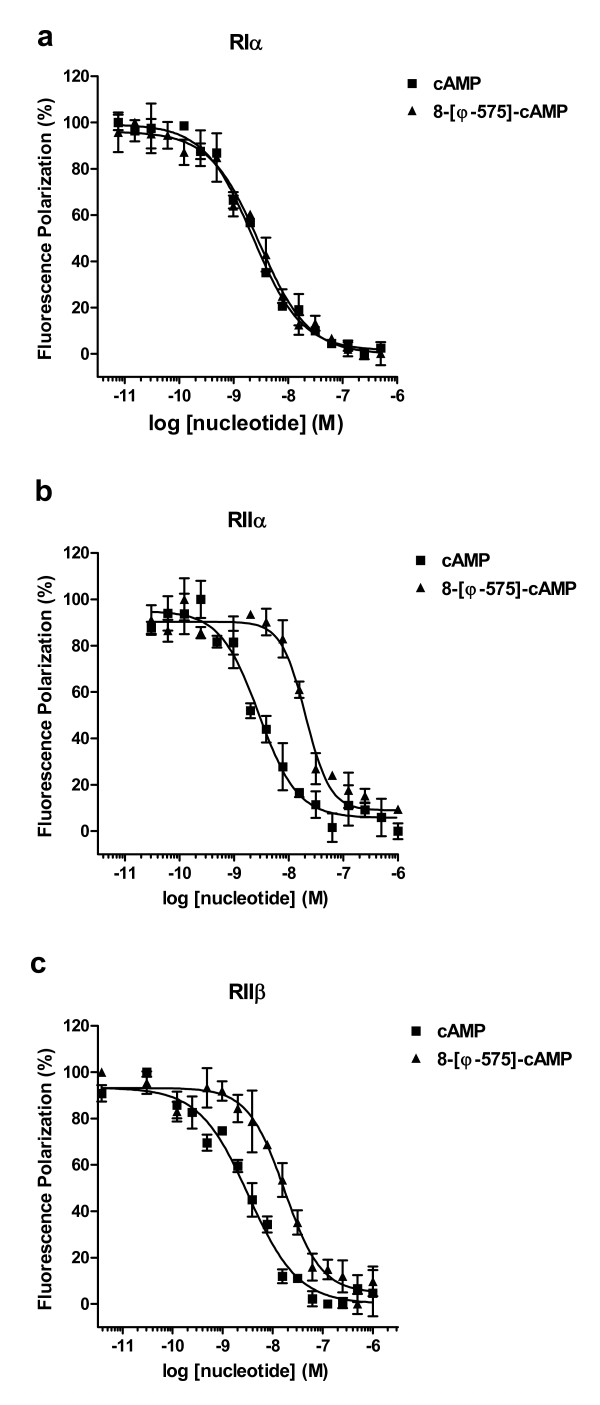
**Competitive nucleotide binding assay using fluorescence polarization**. Serial dilutions of cAMP or 8-[ϕ-575]-cAMP in the presence of 1 nM 8-Fluo-cAMP were prepared. 2.5 nM R subunit was added and fluorescence polarization was determined at Ex485 nm/Em535 nm. EC_50 _values for cAMP and 8-[ϕ-575]-cAMP binding to the R subunit isoforms were deducted from the corresponding titration curves. Each data point represents the mean +/- S.E.M. from at least triplicate measurements.

To investigate the binding of 8-[ϕ-575]-cAMP to RI and RII subunits, we employed a fluorescent polarization displacement assay using 2.5 nM R subunit. Displacement of 8-Fluo-cAMP bound to the R subunits was followed by allowing either cAMP or 8-[ϕ-575]-cAMP to compete with 8-Fluo-cAMP binding. In the case of RIα, we found 8-[ϕ-575]-cAMP and cAMP bound equally well to the R subunit (Fig. 3a). In case of RIIα and RIIβ, cAMP was about 10 times and 5 times more efficient in displacing 8-Fluo-cAMP compared to 8-[ϕ-575]-cAMP, respectively (Fig. 3b und 3c). With respect to both, isoform and site selectivity, and considering the bulky substituent in position 8 of the adenine nucleobase, it could have been expected that 8-[ϕ-575]-cAMP prefers the B- site of PKA type II. However, our data show that binding to RIα is comparable to cAMP, whereas a lower affinity of 8-[ϕ-575]-cAMP for RII isoforms was detected. In this respect, 8-[ϕ-575]-cAMP acts similar to 8-Fluo-cAMP, which shows rather high affinity for the site BII along with rather lower affinity to AII, but a quite equal binding capacity to both sites A and B of RI, thus resulting in an overall higher binding to RIα [23], analogous to other 8-substituted analogs [24,25].

**Figure 4:**
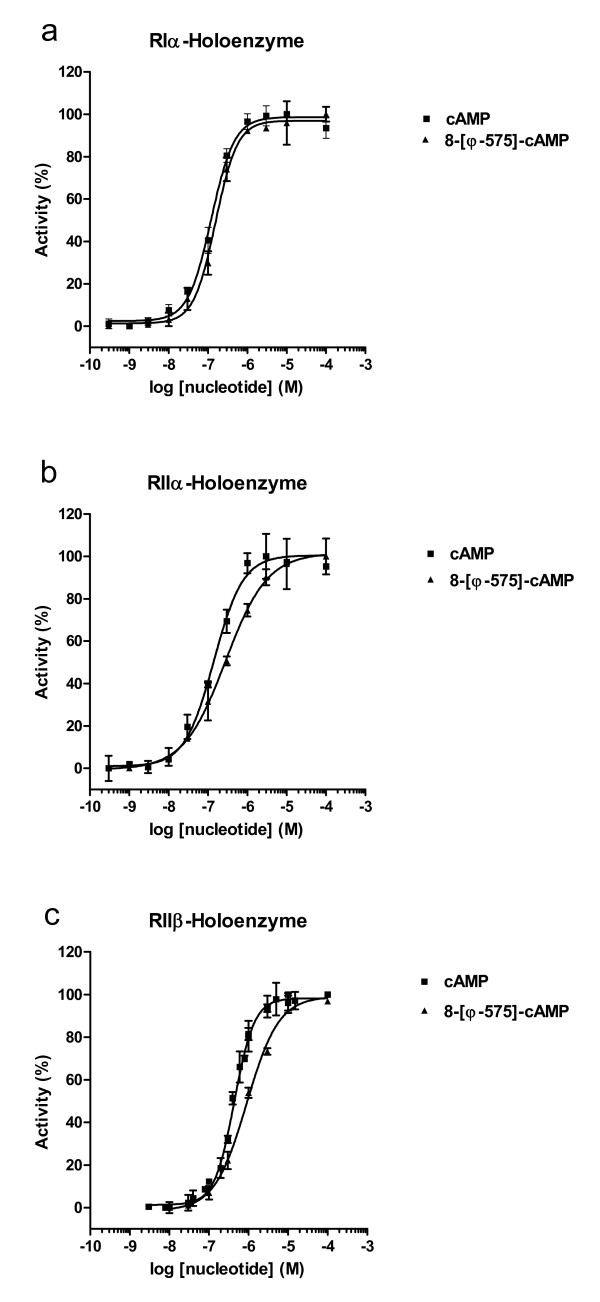
**8-[ϕ-575]-cAMP is a potent PKA activator *in vitro***. For determination of apparent activation constants, purified recombinant R subunits were allowed to form holoenzyme complexes with PKA-Cα (20 nM) as detailed in the methods section. Activation assays were performed by increasing (0.3 nM-10 μM) cAMP. To obtain apparent activation constants (K_act_), the normalized activity of PKA-Cα was plotted against the logarithm of the cAMP (■) and 8-[ϕ-575]-cAMP (▲) concentration and fitted according to a sigmoid dose-response model (Graphpad Prism, variable slope). Each data point represents the mean ± S.D. of two measurements. Experiments were repeated two to three times with similar results.

We next investigated the ability of 8-[ϕ-575]-cAMP to activate PKA-I and -II holoenzymes side by side. RIα, RIIα and RIIβ each were allowed to form a holoenzyme complex with the Cα subunit, before adding increasing amounts of either cAMP or 8-[ϕ-575]-cAMP to re-activate the Cα-subunit. Kinase activity was assayed spectrophotometrically [26], using the synthetic heptapeptide Kemptide as a substrate. The binding properties of 8-[ϕ-575]-cAMP to the RIα subunit is reflected in a nearly identical activation titration curve and corresponding activation constant comparing analog (EC_50 _= 150 nM) with cAMP (EC_50 _= 120 nM, [27,28], Fig. 4a). However, activation of RIIα and RIIβ holoenzymes was less cooperative using 8-[ϕ-575]-cAMP, indicated by a more shallow hill slope of the activation titration curves (Fig. 4a and 4b). The corresponding apparent activation constants (EC_50_-values) were about two-fold increased for 8-[ϕ-575]-cAMP compared to cAMP (EC_50 _RIIα = 280 nM; EC_50 _RIIβ = 900 nM).

### Uptake of 8-[ϕ-575]-cAMP in living cells and activation of PKA

**Figure 5:**
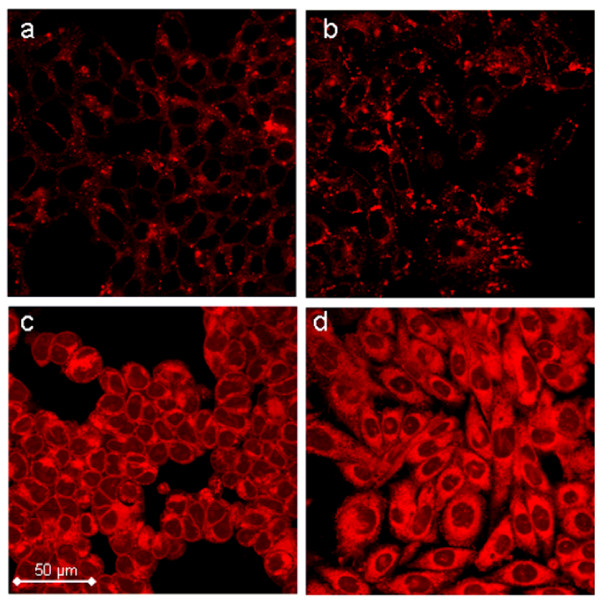
**Visualization of compounds in living cells**. Intracellular imaging of 8-[ϕ-575]-cAMP (a-b) and of Pharos dye (c-d) in HEK293 (a,c) and in CHO (b,d) cells after 1 hour of treatment.

Insights into the intracellular distribution within eukaryotic cells were obtained by intracellular imaging of the cyclic nucleotide analog. 8-[ϕ-575]-cAMP clearly enters the cultivated HEK293 and CHO cells. In most cases it displays a spotty distribution inside the cells and seems to accumulate in bright aggregates over a diffusely labeled background. The nuclear compartment was unlabeled in most cells (Fig. 5a and 5b). In some experiments, employing COS-7 cells less than 5% of the cells showed an accumulation of the compound in the nucleus, which might be attributable to apoptotic degeneration of the cells (data not shown). Variations in the incubation procedure (e.g. decrease in temperature, addition of pluronic^® ^or serum during incubation [29,30]) did not significantly change the distribution pattern or the accumulation in bright spots. However, an incubation temperature of 4°C led to a markedly reduced uptake and accumulation of the compound (data not shown). This could indicate that membrane trafficking, which is inhibited at 4°C, is part of the accumulation process. Whether pinocytotic uptake of the compound or exocytotic processes involving cAMP are inhibited [31], deserves further study. The free Pharos dye efficiently diffuses within the cells and does not accumulate in spots like 8-[ϕ-575]-cAMP does (Fig. 5c and 5d).

**Figure 6:**
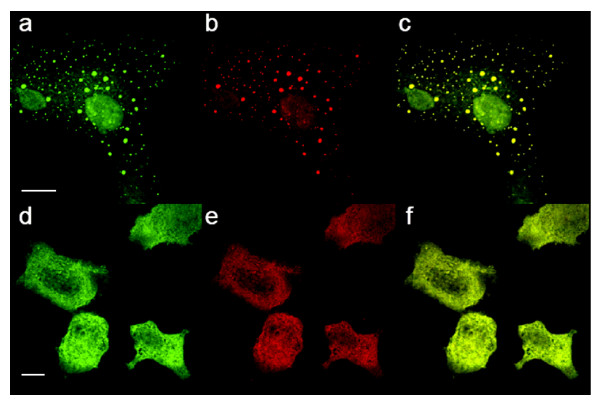
**Co-localization of 8-[ϕ-575]-cAMP and GFP-tagged R subunits**. COS-7 cells were transiently transfected with GFP-hRIα (a-c) and GFP-hRIIα (d-f), incubated with 500 μM 8-[ϕ-575]-cAMP for 30 minutes. Cells were fixed and fluorescence was imaged using confocal microscopy: (a,c) green fluorescence of GFP (b,e), red fluorescence of 8-[ϕ-575]-cAMP, (c,f) merged images. The scale bar indicates 10 μm.

It has previously been shown that fluorescent cAMP analogs were able to label RIα aggregates, respectively [32-34]. We therefore investigated whether the compound co-localizes with GFP-tagged RIα and RIIα proteins expressed in COS-7 cells, and indeed we found co-localization of 8-[ϕ-575]-cAMP, but not the free dye, with R subunits (Fig. 6, and data not shown), indicating that the accumulation of the fluorescent cAMP might be in part due to association to (clustered) cAMP binding proteins. However, we can not entirely exclude unspecific aggregation of 8-[ϕ-575]-cAMP.

In contrast to many other modern fluorophores, the Pharos chromophore has considerably reduced bulkiness along with high lipophilicity. Thus, 8-[ϕ-575]-cAMP has a lower molecular weight compared to e.g. fluorescein-modified-cAMP (8-[2-[(Fluoresceinylthioureido) amino]ethyl]thio]adenosine-3', 5'-cyclic monophosphate; 8-Fluo-cAMP). Due to its merely hydrophobic character, the dye has a big impact on the analog overall lipophilicity, which in turn should compensate the negative charge of the cyclic phosphate and finally lead to good membrane permeability of 8-[ϕ-575]-cAMP. In lipophilicity measurements using reversed phase HPLC [35], its logK_w _was determined to be 2.95 resulting in a more than 70 times increased lipophilicity compared to cAMP (data not shown). In this respect the analog resembles highly membrane-permeant PKA agonists such as Sp-5,6-DCl-cBIMPS [36] or the Epac activator 8-pCPT-2'-O-Me-cAMP [37], and surpasses all fluorescent cAMP analogs described so far. We therefore investigated the kinetics of 8-[ϕ-575]-cAMP uptake in living cells using a genetically encoded fluorescent indicator (H30) formed by a cAMP binding domain from Epac and two spectral variants of the green fluorescent protein (GFP) [38]. Its functioning is based on the phenomenon of fluorescence resonance energy transfer (FRET). FRET relies on a non-radiative, distance-dependent transfer of energy between the two fluorescent domains: by exciting the first, the emission from the second one can be detected. Since FRET depends on the distance between the two fluorophores which in turn is ligand-dependent, this probe is used to estimate the intracellular level of cAMP or -analogs.

**Figure 7:**
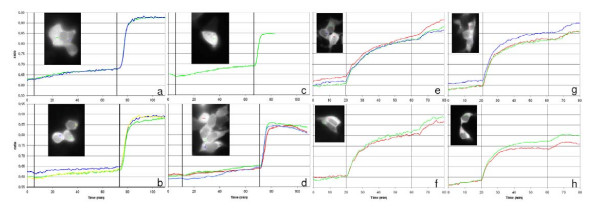
**Uptake of 8-[ϕ-575]-cAMP in intact cells**. Kinetics of 8-[ϕ-575]-cAMP (a-d) and of Sp-5,6-DCI-cBIMPS (e-h) uptake in living cells. HEK293 cells were transfected for transient expression of the H30 indicator of cAMP and treated with 100 μM IBMX to inhibit phosphodiesterases. The ratio between the background subtracted emission intensities at 480 nm and 545 nm is plotted as a function of time. The vertical bars indicate the administrations of either 500 μM 8-[ϕ-575]-cAMP (a-d) or Sp-5,6-DCl-cBIMPS (e-h) and of 25 μM Forskolin, which activates adenylyl cyclase and saturates the FRET-based probe. The insets show the yellow fluorescent protein fluorescence at the beginning of the experiments and the regions of interest where FRET ratios are calculated.

The cells were treated with 500 μM 8-[ϕ-575]-cAMP for one hour and, during this period, FRET ratio increased linearly (Fig. 7a–d). On the basis of the cAMP dose-FRET response curve [39], we estimate that about 7 μM 8-[ϕ-575]-cAMP is available to the intracellular fluorescent indicator. The same experiments were performed using Sp-5,6-DCl-cBIMPS, a highly membrane permeable cAMP analog [36]. In this case, after 40 minutes of treatment, the fluorescent probe was almost completely saturated (Fig. 7e–h), indicating that Sp-5,6-DCl-cBIMPS crosses the plasma membrane more efficiently compared to 8-[ϕ-575]-cAMP, in contrast to the retention based lipophilicity measurements on HPLC, where the analogs behaved more similarly. We can not exclude, however, that the Epac- based FRET sensor used for the measurements preferentially binds Sp-5,6-DCl-cBIMPS.

As the reduced intracellular availability of 8-[ϕ-575]-cAMP could also be due to intracellular PDE-mediated degradation, we tested stability of the compound towards a cAMP-specific PDE isoform (PDE4D5) *in vitro *using a coupled spectrophotometric activity assay [40]. No degradation of 8-[ϕ-575]-cAMP was detected during the 15 minutes of assay duration (data not shown). The specific activity of the enzyme was 0.6 U/mg for cAMP. It has been demonstrated before, that 8-modified cAMP analogs possess considerably high resistance towards PDE degradation [41]. Absolutely stable analogs would arise from conjugating the Pharos fluorophore to phosphorothioate- modified structures such as Rp- or Sp-cAMPS (Rp- or Sp-diastereomer of adenosine 3'-5'-monophosphorothiorate).

**Figure 8:**
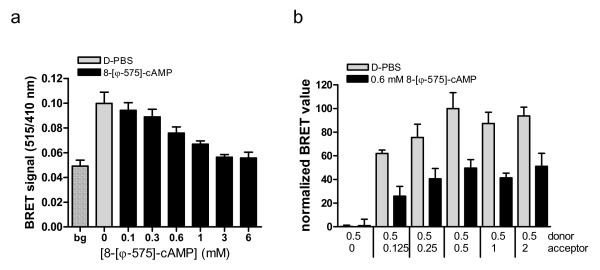
**8-[ϕ-575]-cAMP activates intracellular PKA**. (a) For standard BRET experiments, COS-7-cells were co-transfected with the PKA type II sensor construct or negative control plasmids (bg) as indicated and grown for 48 hours. Cells were treated with the indicated amount of 8-[ϕ-575]-cAMP for 30 minutes, or mock treated (D-PBS). BRET signals were obtained after addition of the luciferase substrate DeepBlueC™ and detection of luciferase and fluorescence light emission using a multi-label reader. Shown is a representative experiment, repeated three times; data are mean ± S.E.M., performed with n = 6 replicates. (b) A BRET titration experiment was performed as described in the methods section. Briefly, cells were co-transfected with a constant amount of BRET donor (hRIIα-Rluc) and an increasing amount of acceptor DNA (GFP^2^-hCα) as indicated. Before BRET read-out, cells were incubated with 0.6 mM 8-[ϕ-575]-cAMP as described above. The BRET values of two independent experiments, each performed with n = 6 replicates, were background subtracted, normalized and plotted as mean ± S.E.M.

We next tested if 8-[ϕ-575]-cAMP can activate the PKA holoenzyme in living COS-7 cells using a recently established bioluminescence resonance energy transfer (BRET) based reporter for PKA-IIα, based on transient co-expressed luciferase-tagged RIIα and (RIIα-RLuc) as the BRET donor and a GFP-tagged Cα subunit (GFP^2^-Cα) as the BRET acceptor. In this assay, a decrease in BRET signal indicates intracellular dissociation of the PKA holoenzyme [42]. As depicted in figure 8a, the cAMP analog efficiently allows the PKA subunits to dissociate. Half-maximal holoenzyme dissociation was achieved at a concentration of about 600 μM analog added to the cells. This value in the same range as the value for e.g. 8-Br-cAMP (EC_50 _= 1.5 mM [42]) but is surpassed by acetoxymethyl esters of cyclic nucleotides, that activate PKA in the low μM range determined by BRET [7] as well as in physiological assays [43]. Finally, we performed a bystander BRET test [44], where COS-7 cells were transfected with a constant amount of donor-expression plasmid (RIIα-RLuc, 0.5 μg) and with increasing amounts of acceptor-expression plasmid (GFP2-Cα, 0–2 μg). The cells were incubated with 600 μM 8-[ϕ-575]-cAMP for 30 minutes prior to the BRET ratio determination, or mock treated. Figure 8b depicts the normalized BRET-values of two experiments each performed with n = 6 wells per incubation. When no BRET acceptor is expressed in the cells, we observed the background BRET value, and the incubation with 8-[ϕ-575]-cAMP has no effect. With increasing amounts of acceptor DNA, the BRET values rises and reaches an asymptote at the 1:1 molar ratio at 0.5 μg donor and acceptor coding DNA each, as expected from a 1:1 interaction of PKA subunits in the holoenzyme. Thereafter the BRET value does not increase further, indicating a specific interaction, which can be prohibited to about 50% by incubation with 600 μM 8-[ϕ-575]-cAMP, as determined previously (see figure 8a and 8b).

## Conclusion

We can conclude that 8-[ϕ-575]-cAMP with its modern, red-shifted fluorophore is a useful, stable tool for *in vitro *and *in vivo *investigation of cAMP binding proteins. It spontaneously enters eukaryotic cells and can be sequestered to PKA regulatory subunits. However, undesired partial accumulation in the cell could not be entirely excluded in this study. It efficiently binds to and activates PKA-I and -II, and should have great potential for many cell biological and *in vitro *applications.

## Methods

### Materials

All cyclic nucleotides, as well as the free Pharos chromophore were derived from Biolog Life Science Institute (Bremen, Germany). The solubility of the novel Pharos compound is approximately 50 mM in water. LogK_w _data were determined by a retention-based lipophilicity ranking using a LiChrograph HPLC (Merck-Hitachi, Darmstadt, Germany) equipped with a reversed phase YMC RP-18 phase (250 × 4 mm) running at a flow rate of 1.0 ml/min. Nucleotides were detected at λ = 280 nm [35]. A. The purity of cyclic nucleotides was analyzed with an elution system consisting of 25% acetonitrile and 10 mM triethyl ammonium formiate at a flow rate of 1.5 ml/min and was found to be > 99%. No trace of free fluorophore was detected in 8-[ϕ-575]-cAMP.

### Spectral measurements

The Pharos dye was diluted in 20 mM 2-(4-morpholino)-ethane sulfonic acid (MES) buffer adjusted to pH 6.0, pH 7.0 and pH 7.4, and all samples were degassed by bubbling nitrogen through the solution before measuring the absorption or fluorescence spectra.

For Stokes shift and quantum yield determination, absorption and fluorescence spectra were recorded in cells with 1 cm path length using a PerkinElmer Lambda 900 UV-Vis spectrophotometer and a Hitachi F-4500 fluorescence spectrophotometer, respectively.

To calculate the relative quantum yield ϕ of the free Pharos dye, the absorption (500 nm to 640 nm) and fluorescence (λ_ex _= 553 nm, λ_em _ranging from 558 nm to 800 nm) spectra of several Pharos dye concentrations (640 nM to 64.2 μM) were recorded in A. bidest. The integrated fluorescence intensity (I) was plotted versus the absorbance (A) at the excitation wavelength. For low concentrations the dye molecules are not influenced by each other and thus exhibit a linear behavior concerning absorption and emission of light. Linear regression yields the slope ΔI/ΔA that is compared to the corresponding data of a quantum yield standard by using the equation ϕ_sa _= (ΔI_sa_/ΔA_sa_)·(ΔA_st_/ΔI_st_)·ϕ_st_. As a standard, a solution of quinine sulfate in 1.0 N sulfuric acid (ϕ_st _= 0.546 with λ_ex _= 365 nm) was used [21,22].

For emission and excitation spectra (Kontron SFM25) with and without purified regulatory subunit of PKA (see below), 100 nM 8-[ϕ-575]-cAMP was measured with and without fourfold molar excess of regulatory subunit in 20 mM MOPS, 150 mM NaCl, 1 mM β-mercaptoethanol, pH (buffer A) at room temperature. The excitation spectra were detected at λ_em _= 617 nm with λ_ex _ranging from 610 nm to 430 nm; emission spectra were measured with λ_ex _= 575 nm with λ_em _ranging from 800 nm to 580 nm.

### Expression and purification of PKA subunits

For expression of PKA regulatory subunits, one liter of Luria Broth medium containing 100 μg/ml of ampicillin was inoculated with *E. coli *BL21 (DE3) Codon Plus RIL cells (Stratagene) transformed with human RIα, RIβ, RIIα (in pRSET_B_) or rat RIIβ (in pETIIc) and grown at 37°C to an OD_600 _nm of 0.8. Recombinant protein expression was induced by addition of 0.2 M isopropyl-β-D-thiogalactopyranoside (IPTG) and the culture was incubated at 25°C for additional 17–18 h. The pellets were stored at -20°C.

For purification of RI isoforms (Moll et al., submitted), cell lysis of protein expressing *E. coli *cells was performed two times with a French Pressure Cell (Thermo Electron Corp., Needham Heights, USA) in lysis buffer (20 mM 3-(N-morpholino) propane sulfonic acid (MOPS), 100 mM NaCl, 1 mM β-mercaptoethanol, 2 mM EDTA, 2 mM EGTA, pH7.0). The lysate was centrifuged at 27 000 ×g for 30 min and 4°C. 1.2 μmol Sp-8-AEA-cAMPS-agarose (Biolog Life Science Inst.) was used per purification, corresponding to 300–450 μl agarose-slurry. The protein content in 12 ml clarified supernatant was batch bound by gentle rotation over night by 4°C. The agarose was washed seven times with 1.25 ml lysis buffer. The protein elution step was performed with 1.25 ml of 10 mM cGMP in buffer A by gentle rotation at 4°C for 1 h. The agarose was rinsed with two additional wash steps (each 825 μl) with buffer A. Subsequently, the R subunits were subjected to gel filtration (PD10, Amersham Pharmacia Biotech, Freiburg, Germany) into buffer A. To remove all cGMP, the R subunits were dialyzed excessively against buffer A.

For purification of RII isoforms (Moll et al., submitted), cell lysis was performed in buffer consisting 20 mM MES, pH6.5, 100 mM NaCl, 5 mM EDTA, 5 mM EGTA and 5 mM β-mercaptoethanol with added protease inhibitors (PI): Leupeptin (0.025 mg/100 ml), TPCK and TLCK (each 1 mg/100 ml) (buffer B). after centrifugation at 27 000 ×g for 30 min and 4°C, the supernatant was precipitated at 4°C with 50% saturated ammonium sulfate (AS) for RIIα and 45% AS for RIIβ and centrifuged by 10 000 ×g, 15 min 4°C. The AS pellets were re-suspended in buffer B and protein was batch bound to 1.4 μmol settled Sp-8-AEA-cAMPS-agarose. The agarose was rinsed twice with 20 mM MOPS, pH7.0, 1 M NaCl, 5 mM β-mercaptoethanol and then two times with 10 ml buffer B. Subsequently, two elution steps were carried out with 25 mM cGMP in buffer B. RII subunits were subjected to gel filtration into 20 mM MES, pH 6.5,150 mM NaCl, 2 mM EDTA, 2 mM EGTA, 1 mM β-mercaptoethanol.

Murine Cα subunit (in pRSET_B_) was expressed in *E. coli *BL21 (DE3) (Stratagene) and purified as published previously [45,46]. Protein expression and purification was followed by SDS-polyacrylamid gel electrophoresis [47]. Typically, the recombinant proteins were purified to 95% homogeneity or higher.

### Fluorescence Polarization (FP)

The fluorescence polarization displacement assay was performed as described before[7]. Increasing concentrations of cAMP or 8-[ϕ-575]-cAMP were mixed with 1 nM 8-Fluo-cAMP before adding 2.5 nM regulatory subunit RIα, RIIα, RIIβ. Fluorescence polarization was measured after 5 minutes of incubation at room temperature.

### Determination of activation constants

PKA activity was assayed by the coupled spectrophotometric assay first described by Cook et al. [26] using 260 μM Kemptide (LRRASLG) as the substrate. Holoenzyme formation was carried out for 3 minutes at room temperature with 20 nM murine PKA-Cα subunit and an about 1.2 fold molar excess cAMP-free RIα, RIIα, or RIIβ subunit in assay mixture (10 mM MgCl_2_, 100 μM ATP, 100 mM MOPS, 1 mM PEP, LDH, pyruvate kinase, NADH, 5 mM β-mercaptoethanol, pH 7.0). Apparent activation constants (K_act_, EC_50_) were determined by adding increasing amounts of cAMP or 8-[ϕ-575]-cAMP (0.3 nM to 10 μM).

### BRET assay

COS-7 cells were used for BRET experiments. They were routinely passaged and seeded in opaque 96-well microplates (CulturPlate™-96, PerkinElmer) 24 hours prior to co-transfection with the previously described PKA-IIα sensor, comprised of RIIα-RLuc (donor) and GFP2-Cα (acceptor) [42]. Two days following transfection with 0.5 μg donor and acceptor DNA, respectively, cells were rinsed with glucose-supplemented Dulbecco's PBS (D-PBS, Invitrogen), and subsequently incubated with 8-[ϕ-575]-cAMP (0.01–6 mM final concentration in D-PBS, prepared from a 20 mM stock solution), or mock treated for 30 minutes at room temperature. For the BRET read-out, the luciferase substrate DeepBlueC™ (PerkinElmer) was added at a final concentration of 5 μM in a total volume of 50 μl D-PBS. Light output was detected consecutively using a Fusion™ α-FP microplate reader (PerkinElmer, read time 1s, gain 25) equipped with appropriate filters for the donor (RLuc; λ = 410 nm ± 80 nm) and for the acceptor fluorophore (GFP^2^; λ = 515 nm ± 30 nm) emission. Emission values obtained with untransfected (n.t.) cells were routinely subtracted, and BRET signals were calculated as follows: (emission_(515nm) _– n.t. cells_(515nm)_)/(emission_(410nm) _– n.t. cells_(410nm)_). Control measurements with cells expressing RLuc and GFP proteins without a fusion partner yield the background BRET signal. A BRET titration (bystander BRET test) was performed by co-transfection of COS-7 cells with a constant donor-expression plasmid (0.5 μg) with an increasing amount of acceptor-expression plasmid (0–2 μg). The cells were treated as described above. Prior to the BRET read-out, cells were incubated with or without 0.6 mM 8-[ϕ-575]-cAMP for 30 minutes.

### Uptake of 8-[ϕ-575]-cAMP in living cells

HEK293 and CHO cells were grown in the Dulbecco's modified Eagle medium (DMEM, Invitrogen) and F12 nutrient mixture (HAM, Invitrogen) mediums, respectively, containing 10% FBS and supplemented with 2 mM L-glutamine, 100 U/ml penicillin, and 100 μg/ml streptomycin (all: Sigma-Aldrich), in a 37°C humidified atmosphere containing 5% CO_2_. These cell lines are routinely used in our laboratory for FRET experiments using various sensors and they have been thoroughly characterized for fluorescent sensor expression levels.

For transient expression of the H30 sensor, cells were seeded onto 24-mm diameter round glass coverslips, and transfections were performed at 50–70% confluence with FuGENE-6 transfection reagent (Roche). Imaging experiments were performed after about 24 h. Cells were maintained in Hepes-buffered Ringer-modified solution, containing 125 mM NaCl, 5 mM KCl, 1 mM Na_3_PO_4_, 1 mM MgS0_4_, 5.5 mM glucose, 1 mM CaCl_2_, and 20 mM Hepes, pH 7.4, at room temperature and treated with 100 μM 3-Isobutyl-1-methylxanthine (IBMX, Sigma-Aldrich) 10 minutes before the experiments. Cells were imaged on an inverted microscope (IX50; Olympus) with a 60× oil immersion objective (Olympus). The microscope was equipped with a monochromator (Polychrome IV; TILL Photonics) and a beam-splitter optical device (Multispec Microimager; Optical Insights). FRET variations were measured as changes of the ratio between the background-subtracted fluorescence emission intensities at 480 nm and 545 nm, on excitation at 430 nm. Forskolin (25 μM, Sigma-Aldrich) was added to saturate the FRET probe and to determine the maximal FRET response.

For the imaging of the intracellular distribution of 8-[ϕ-575]-cAMP and the Pharos dye, cells were grown and seeded as above. Before the image acquisitions, cells were treated with 100 μM IBMX and either 500 μM 8-[ϕ-575]-cAMP or the Pharos dye for 1 hour. Cells were then washed with the Hepes-buffered Ringer-modified solution described above and imaged on a confocal microscope (Leica) with a 20× oil immersion objective (Leica). Images were obtained by collecting the emission light from 600 nm to 640 nm, on excitation at 514 nm.

Intracellular co-localization of 8-[ϕ-575]-cAMP and GFP-hRIα or GFP-hRIIα was examined in COS-7 cells after two days transient expression of the GFP-tagged R subunits [42]. Cloning of the R subunits into the expression vector hpGFP^2^-N_2 _(PerkinElmer) was performed as described previously [48]. The cells were incubated for 30 minutes at 37°C with 500 μM 8-[ϕ-575]-cAMP in D-PBS, rinsed tree times with D-PBS, followed by a standard fixation procedure [42]. Cellular imaging was performed on a confocal microscope (Leica) with a Plan apo 100× oil immersion objective (Leica).

### Statistical procedures

Measurement and statistical evaluation of FP, kinase activity and BRET assays was carried out using GraphPad Prism software version 4 (GraphPad Software).

## Abbreviations

8-[ϕ-575]-cAMP: 8-[Pharos-575]adenosine -3',5'-cyclic monophosphate; 8-Fluo-cAMP: 8-[2-[(Fluoresceinylthioureido)amino]ethyl]thio]adenosine -3',5'-cyclic monophosphate; Sp-5,6-DCl-cBIMPS: 5,6-dichlorobenzimidazole riboside -3',5'-cyclic monophosphorothioate, Sp-isomer; 8-pCPT-2'-O-Me-cAMP: 8-(4-chlorophenylthio)-2'-O-methyladenosine -3',5'-cyclic monophosphate; 8-Br-cAMP: 8-Bromoadenosine -3',5'-cyclic monophosphate; Sp-8-AEA-cAMPS-agarose: 8-(2-aminoethylamino)adenosine -3',5'-cyclic monophosphorothioate, Sp- isomer, immobilized to agarose; MOPS: 3-(N-morpholino) propane sulfonic acid; MES: 2-(4-morpholino)-ethane sulfonic acid; PKA: protein kinase A, cAMP-dependent protein kinase; R: regulatory subunit; C: catalytic subunit; FRET: Fluorescence resonance energy transfer; BRET: bioluminescence resonance energy transfer; PDE: phosphodiesterase; IBMX: 3-Isobutyl-1-Methylxanthine.

## Authors' contributions

**H–GG **synthesized the Pharos compounds and performed studies on stability and lipophilicity, **DM **and **CMB **performed the physical characterization experiments, **DM **performed the FP assay, **AP **coordinated the study, performed BRET and kinase activity assays, cellular distribution evaluation and co-localization experiments; **MB **performed FRET assays and cellular distribution evaluation, **KG **performed cellular co-localization experiments and the PDE assay, **FWH **and **MZ **contributed to the design of the experiments in this study and the data evaluation, **AP **and **H–GG **wrote the manuscript.

## Supplementary Material

Additional File 1 An additional PDF-document (Moll_Prinz_et al_Additional file) illustrating the quantum yield determination of the Pharos dye is provided. Click here for file
