# Supplementary material for: Biochemical characterization and cellular imaging of a novel, membrane permeable fluorescent cAMP analog
Source: BMC Biochem. 2008 Jun 25;9:18. doi: 10.1186/1471-2091-9-18 (PMC2443153; doi:10.1186/1471-2091-9-18)
Supplement: Additional File 1 — An additional PDF-document (Moll_Prinz_et al_Additional file) illustrating the quantum yield determination of the Pharos dye is provided. [file 1471-2091-9-18-S1.pdf]

a

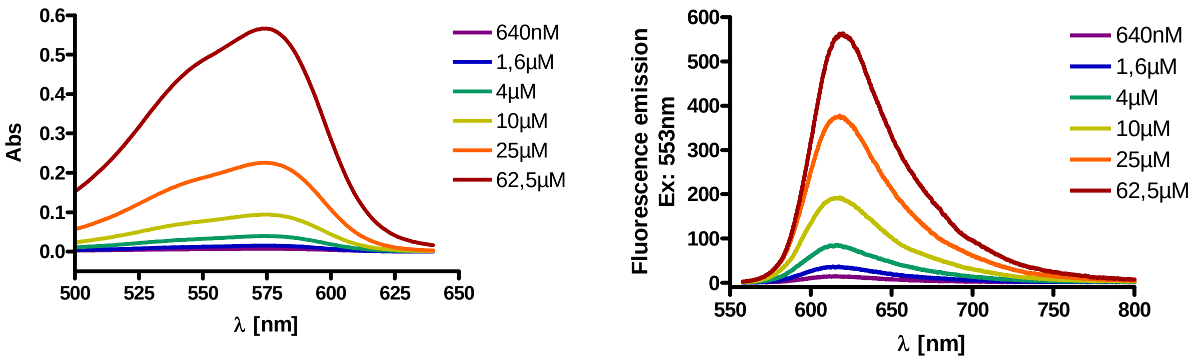

b

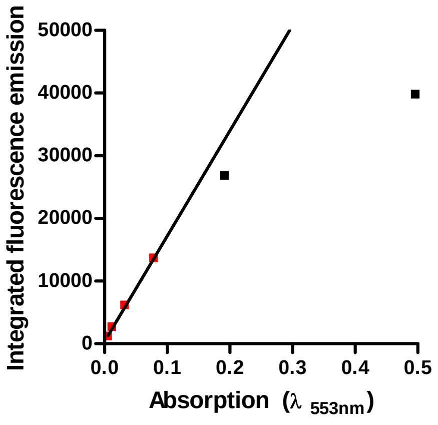

(a) Absorption and emission fluorescence spectra for quantum yield determination at different concentrations of the free Pharos dye. (b) Plotting the integrated fluorescence intensity versus the corresponding absorption value for quantum yield determination: For low concentrations when molecules are not influenced by each other there is a linear relation (red squares). The slope of the linear regression was compared to the standard substance quinine sulphate to calculate the relative quantum yield.
